# Endoscopic versus open radial artery harvest and mammario-radial versus aorto-radial grafting in patients undergoing coronary artery bypass surgery: protocol for the 2 × 2 factorial designed randomised NEO trial

**DOI:** 10.1186/1745-6215-15-135

**Published:** 2014-04-23

**Authors:** Christian L Carranza, Martin Ballegaard, Mads U Werner, Philip Hasbak, Andreas Kjær, Klaus F Kofoed, Jane Lindschou, Janus Christian Jakobsen, Christian Gluud, Peter Skov Olsen, Daniel A Steinbrüchel

**Affiliations:** 1 Department of Cardio-thoracic Surgery, The Heart Centre, Rigshospitalet, Copenhagen University Hospital, Copenhagen, Denmark; 2 Department of Clinical Neurophysiology, Rigshospitalet, Copenhagen University Hospital, Copenhagen, Denmark; 3 Multidisciplinary Pain Centre, Rigshospitalet, Copenhagen University Hospital, Copenhagen, Denmark; 4 Department of Clinical Physiology, Nuclear Medicine and PET, Rigshospitalet, Copenhagen University Hospital, Copenhagen, Denmark; 5 Department of Cardiology and Radiology, Rigshospitalet, Copenhagen University Hospital, Copenhagen, Denmark; 6 Copenhagen Trial Unit, Centre for Clinical Intervention Research, Rigshospitalet, Copenhagen University Hospital, Blegdamsvej 9, 2100 Copenhagen, Denmark

**Keywords:** Arterial revascularisation, Coronary artery bypass, Endoscopic radial artery harvest, Nerve conduction study, Neurological deficit, Open radial artery harvest, Patency, Quantitative sensory testing, Radial artery harvest

## Abstract

**Background:**

Coronary artery bypass grafting using the radial artery has, since the 1990s, gone through a revival. Observational studies have indicated better long-term patency when using radial arteries. Therefore, radial artery might be preferred especially in younger patients where long time patency is important. During the last 10 years different endoscopic techniques to harvest the radial artery have evolved. Endoscopic radial artery harvest only requires a small incision near the wrist in contrast to open harvest, which requires an incision from the elbow to the wrist. However, it is unknown whether the endoscopic technique results in fewer complications or a graft patency comparable to open harvest. When the radial artery has been harvested, there are two ways to use the radial artery as a graft. One way is sewing it onto the aorta and another is sewing it onto the mammary artery. It is unknown which technique is the superior revascularisation technique.

**Methods/Design:**

The NEO Trial is a randomised clinical trial with a 2 × 2 factorial design. We plan to randomise 300 participants into four intervention groups: (1) mammario-radial endoscopic group; (2) aorto-radial endoscopic group; (3) mammario-radial open surgery group; and (4) aorto-radial open surgery group.

The hand function will be assessed by a questionnaire, a clinical examination, the change in cutaneous sensibility, and the measurement of both sensory and motor nerve conduction velocity at 3 months postoperatively. All the postoperative complications will be registered, and we will evaluate muscular function, scar appearance, vascular supply to the hand, and the graft patency including the patency of the central radial artery anastomosis. A patency evaluation by multi-slice computer tomography will be done at one year postoperatively.

We expect the nerve conduction studies and the standardised neurological examinations to be able to discriminate differences in hand function comparing endoscopic to open harvest of the radial artery. The trial also aims to show if there is any patency difference between mammario-radial compared to aorto-radial revascularisation techniques but this objective is exploratory.

**Trial registration:**

ClinicalTrials.gov identifier: NCT01848886.

Danish Ethics committee number: H-3-2012-116.

Danish Data Protection Agency: 2007-58-0015/jr.n:30–0838.

## Background

### Current treatment

In coronary bypass surgery, a vein or artery graft is used to lead the blood past the stenosis that gives rise to the symptoms of the patient. This graft can be taken from the chest, leg, or arm. The left internal mammary artery (LIMA), which is situated on the inside of the chest wall, is the most commonly used graft. This graft has the best patency and is often used as a bypass to the anterior descending coronary artery (LAD) situated on the front left side of the heart. The saphenous vein from the leg is often the second choice. It is technically easy to harvest and it is long, allowing for multiple grafts. Another possibility is using the radial artery, which might have a better long-term patency than the saphenous vein. It is much shorter in length and therefore it can be difficult to reach all the coronary arteries required for a bypass graft.

The radial artery graft has in some studies shown a short-term patency of about 86% to 91% [1-3] and a long-term patency of about 81% to 89% [3-7]. Also, a lower short-time patency of 81% to 86% has been shown when using the saphenous vein grafts [4,8]. However, one of these studies had conflicting results [8]. In a cohort study of 925 patients, saphenous vein graft recipients had a significantly higher mortality than radial artery graft recipients 0 to 6 years after bypass surgery [9]. Guidelines on myocardial revascularisation recommend using total arterial revascularisation in patients with a substantial post-operative life expectancy [10].

### Clinical data on the experimental interventions

Endoscopic vessel harvesting was first introduced in the mid-1990s, where the procedure was used to harvest the saphenous vein. By 2005, it was recognised as the procedure of choice [11] and it is now used in about 80% of patients at US hospitals. Between 2001 and 2004, three case studies describing endoscopic radial artery harvest (ERAH) were published [12-14].

### Complications after radial artery harvest

The largest case-control study of ERAH reported fewer complications compared to open surgery [15]. Other studies have shown the same, including a significant decrease in other complications such as haematomas and infections [16-18]. So far, only one randomised clinical trial dealing with postoperative neurological deficits and pain has been published [19]. This trial (n = 50) reported fewer neurological complications (11 (44%) in the ERAH group versus 17 (85%) in the open radial artery harvest (ORAH) group; *P* = 0.23) and a significantly lower number of severe neurological complications (score of 1–5 points, 0.8 ± 1.1 in ERAH group versus 2.2 ± 1.2 in ORAH group; *P* < 0.001) when assessed by telephone interviews of self-reported complications.

The reported rate of neurological complications after ORAH is about 11% to 39% [20-23]. The highest prevalence was found in the study by Dick et al., where 39% of the patients had self-reported persistent discomfort at the arm at a mean of 0.9 years postoperatively (numbness (32%), paraesthesia or dysesthesia (14%), pain (5%)) [22]. In three out of the four studies, more than 30% of patients had a self-reported neurological deficit.

ERAH reduced the risk of neurological complications in one prospective cohort study from 10% to 0% [17]. The largest published series of ERAH from Connolly et al. reported a complication rate of about 10%, but the sensitivity to detect complications was limited by the follow-up being done by telephone interview or office visit alone with no objective clinical examination [13]. The published studies differ in how the investigators examine the extent of neurological deficits. Most use non-specific neurological examinations and others only rely on questionnaires [24]. The most specific examination procedure seems to be used by Bleiziffer et al., who assessed the muscle strength, deep tendon reflexes, temperature, pain, and touch sensation [18].

### Patency in endoscopic harvested graft material

Three large observational studies of short-term patency in endoscopic harvested veins has questioned the patency when using the endoscopic technique (EVH) compared to the open technique (OVH) [25-27]. All three studies reported lower vein graft patency and one of them even a higher mortality when using EVH. On the contrary, most other database studies have not shown a higher mortality after EVH than after OVH [28-31]. The latest retrospective cohort study included an unprecedented 235,000 patients and showed no difference in mortality between EVH and OVH at 3-year follow-up [32]. It has been proposed that the possible reasons for a lower patency in EVH might be trauma to the vessel during endoscopic harvest and thermic spread from the ligating device, but most studies of graft histology and function have shown no difference resulting from the techniques used [33].

The patency in ERAH versus ORAH could be questioned. Three studies have dealt with patency differences comparing open surgery with the endoscopic technique. One study appraised the patency assessed by 64 multi-slice CT (MSCT) in 33 patients and by angiography in 17 patients and found no difference [34]. Two other studies included 94 patients undergoing 16 slice MSCT and 95 patients undergoing either angiography or CT-scan, respectively, before discharge [16,35]. Neither of the studies reported a difference in the short-term patency. None of the patency studies have used a randomised clinical trial design. As with the EVH studies, the studies using radial artery grafts have reported no difference in histology or function [36,37].

### Composite grafting

When using the radial artery graft it is possible to anastomose it either directly onto the aorta (aorto-radial/free radial) or onto the side of the LIMA (mammario-radial/Y-graft) as a so-called composite graft. Different techniques of using the radial artery graft as a composite graft are well described [38-40]. One retrospective cohort study showed a higher risk of string-sign and a lower patency when comparing composite grafting with individual grafting [41]. This was only related to target vessels with mild stenosis. Another large retrospective case-control study of 893 patients showed a lower patency in composite grafts compared to direct aortic grafts [42]. Other studies have shown the site of the proximal anastomosis to be without any influence on the resulting patency [43-45]. With composite grafting it is possible to perform a greater number of distal coronary anastomoses and the proximal anastomosis does not require aortic partial clamping, which may increase the risk of cerebral embolism. A retrospective study showed a non-significant difference in patency between composite grafts (T-graft) and free radial grafts [46]. One randomised clinical trial comparing patency after mammario-radial grafting showed a significantly better clinical outcome and better patency in the radial artery composite graft group [47,48]. Another randomised clinical trial (Stand-in-Y Mammary Study) compared the right internal mammary artery as a Y-graft versus radial artery as a free graft and showed a significantly better survival when revascularisation was done with two arteries compared to one artery [49].

### Trial objectives

#### The NEO trial 1

The primary objective will be to assess ERAH versus ORAH on:

• The hand function, assessed by a questionnaire.

The secondary objectives will be to assess ERAH versus ORAH on:

• Postoperative neurological deficits using nerve conduction studies.

• Postoperative neurological deficits using clinical examination of motor function and quantitative sensory testing.

• Differences in complications (ischemia, infections, wound dehiscence, haematoma formation, etc.).

Further, the exploratory objectives will be to assess ERAH versus ORAH on:

• Serious adverse events.

• The postoperative scar.

• Postoperative handgrip strength.

• Postoperative muscle function.

• Hand function questionnaire single items.

• Neurological deficits single test.

• Graft patency.

• Postoperative pain in the donor arm.

• Examination of the vascular supply to the hand after radial artery harvest.

#### The NEO trial 2

The primary objective will be to assess mammario-radial versus aorto-radial grafting on:

• The composite cerebrovascular outcome of cerebral stroke, postoperative revascularisation, myocardial infarction, or all-cause mortality.

The secondary objective will be to assess mammario-radial versus aorto-radial grafting on:

• Graft patency evaluated by MSCT.

## Methods and design

### Design and randomisation procedure

The NEO trial will be a randomised clinical trial using the 2 × 2 factorial trial design with a partly blinded outcome assessment. We assume that there is no interaction between the interventions being assessed.

The Copenhagen Trial Unit will conduct central web-based randomisation. All participants will be randomised in the morning on the day of coronary artery bypass grafting surgery (CABG) surgery. Approximately 150 patients will be allocated to the ERAH group and approximately 150 patients to the ORAH group (the NEO trial 1). The two groups will furthermore be randomised into two groups of 75 patients each. The two groups will differ in how the radial artery is proximally anastomosed to either the aorta or to the LIMA (the NEO trial 2). In all, there will be approximately 150 patients with an aorto-radial anastomosis and approximately 150 patients with a mammario-radial anastomosis. The allocation sequence will be a computer-generated using varying block sizes and will be concealed to the investigators. The randomisation will be stratified by age (age up to 59 years compared to 60 years or older) and sex.

**Figure 1:**
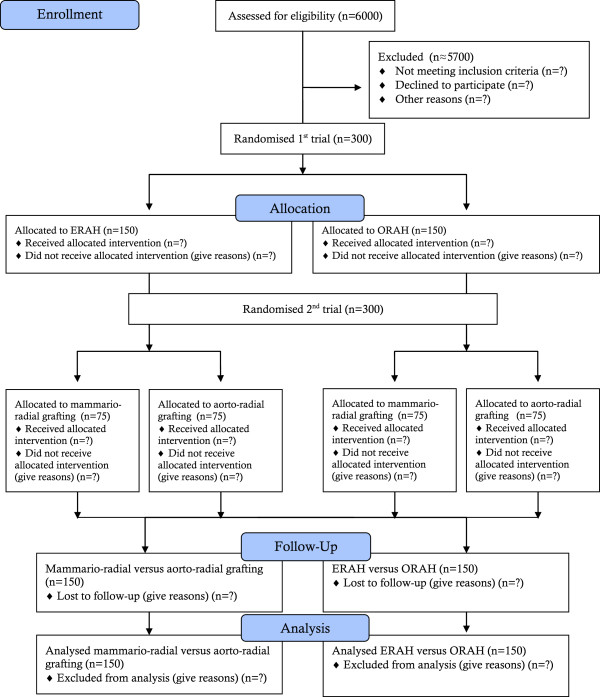
Trial flow chart.

The trial flow chart can be seen in Figure 1.

### Selection of participants

All non-acute patient referrals to the Department of Cardio-thoracic Surgery, Rigshospitalet, University Hospital of Copenhagen, Denmark, will be screened for eligibility. Approximately 6,000 patients will be screened. Patients will be eligible for the NEO trial, if they fulfil the following inclusion criteria and if they do not fulfil any exclusion criteria.

### Inclusion and exclusion criteria

#### Inclusion criteria

• Elective or subacute coronary artery bypass graft as an isolated procedure.

• Age >18 years.

• Coronary multi-vessel disease.

• Non-dominant arm is eligible for radial artery harvest.

• Written informed consent.

#### Exclusion criteria

• Geographically not available for follow-up.

• Modified Allen’s test indicating insufficient ulnary artery perfusion.

• Valve surgery, ablation surgery, or any kind of concomitant surgery during same admission.

• Acute operation (<24 hours from admission).

• Dialysis.

• Preoperative neurological deficit on the donor arm.

• Left ventricular ejection fraction <20% preoperatively.

• Prior sternotomy.

• Allergy to contrast medium.

• Malignant disease.

• No written informed consent.

Preoperatively, all patients will have a modified Allen’s test performed to ensure sufficient vascular function of the ulnary artery. A modified Allen’s test is defined as a normal capillary response in the hand in less than 7 to 10 seconds after relief of digital compression of the ulnar artery during a strong fist grip emptying the hand of blood [50].

### Blinding

The patients and surgeons cannot be blinded to the intervention used, as it will be obvious to both patient and surgeon whether the radial artery has been harvested with endoscopic or open techniques. Likewise, neither the patients nor the surgeons can be blinded to which proximal anastomotic site is used, since the surgeon will be performing the anastomosis and the patient has the right to be informed about the procedure performed.

The neurophysiology technicians cannot be blinded to whether ERAH or ORAH has been used since the neurophysiological examination requires placing electrodes near the scar evidently showing which procedure was used.

The NEO trial nurse cannot be blinded since she will be aware of screening, randomisation, and outcome measurements of the individual patients.

The clinical staff examining handgrip strength and muscle function will be blinded to which intervention has been used.

The staff interpreting the MSCTs cannot be blinded in consideration to the NEO trial 2 but will be blinded in consideration to the NEO trial 1.

The data collection will be blinded when using register data.

The statistical analysis of the trial will be blinded with the intervention groups coded as, e.g., ‘X’ and ‘Y’, following which two conclusions will be drawn: one assuming ‘X’ is the experimental group and ‘Y’ is the control group, and one conclusion assuming the opposite. After this, the blinding will be broken.

As to the specific blinding of outcomes see the section ‘Assessment of outcomes’.

### Trial interventions

The non-dominant arm will always be preferred. This will be determined by self-reporting from the patient. The occurrence of neurological damage in the dominant arm can severely impair the patient’s function postoperatively and therefore harvesting of the radial artery in the dominant arm will not be done.

### The NEO trial 1

The unpublished department routines for operative techniques used on the donor arm are described in Table 1.

**Table 1:** Trial interventions

ORAH	The skin is incised by scalpel making a 3 cm long incision. The radial artery is dissected free and a vascular clamp is positioned across the artery. If saturation in the index finger on the non-dominant hand is unchanged and the pulsatile flow measured by pulse-oximetry is not compromised, the incision is continued from 2 cm proximally to the wrist and all the way to about 4 cm from the fossa cubiti. The radial artery is dissected free from surrounding tissue by scissors. Side branches are localised and divided by electrical cutters (‘Cautery Forceps’ manufactured by Starion Instruments). When the artery is totally free it is ligated and divided at both ends. The incision is closed with Vicryl 3–0 continuous suture in the subcutis and Vicryl 4–0 continuous intradermal suture.
ERAH	A 2 to 3 cm long incision is made over the radial artery at the wrist on the non-dominant arm. As with the open procedure a pulse-oximetry is placed on the arms index finger. The artery is clamped with a vascular clamp thereby insuring the hand is sufficiently perfused from the ulnary artery. The Maquet Haemopro system (manufactured by MAQUET Gmbh & Co. KG) is then used to dissect the artery free by ligating the side-branches using the Haemopro’s scopical ligating forceps. To free the artery proximally a stab incision is made in the fossa cubiti through which the artery is ligated and divided. The incision in the fossa cubiti is only approximated by Steri-strips but the incision near the wrist is closed with Vicryl 3–0 in the subcutis and Vicryl 4–0 intra-dermally.
Mammario-radial graft	A mammario-radial graft (Y-graft) is performed before extracorporeal circulation (ECC) is begun. When the mammary artery has been mobilised and the radial artery harvested, an end-to-side anastomosis is done with the proximal end of the radial artery being attached to the side of the mammary artery. The anastomosis is sewn with a Prolene 7–0 suture. Free flow through the anastomosis is checked and papaverin solution is applied to the LIMA and radial artery grafts. ECC is begun, the cross-clamp is positioned, and cardioplegia is given. The anastomoses of the radial artery to the coronaries are done from the proximal site going distally. After all radial artery anastomoses are done, the LIMA to LAD anastomosis is performed. After measuring flow in the grafts using ultrasound, the ECC is weaned according to department procedure. Closure and the remaining hospital stay also follow department procedures.
Aorto-radial graft	An aorto-radial graft (free radial artery graft) is performed when the radial artery graft is sewn directly onto the aorta ascendens. This is done after all coronary anastomoses have been completed. ECC is still in effect and a sideclamp is positioned on the aorta ascendens where the cardioplegia cannula is placed. The puncture site for the cardioplegia cannula is also used as the proximal anastomosis site. The proximal anastomosis is done using a Prolene 6–0 suture. Air is removed by retrograde de-airing removing the small vascular clamp positioned on the radial artery graft. The cross-clamp is removed after measuring flow in the grafts using ultrasound and ECC is weaned according to department procedure. Closure and the remaining hospital stay also follow department procedures.

After harvest the radial graft will be positioned in a solution of heparin and blood until bypass grafting is done. In both groups all side-branches are clipped with titanium clips. The techniques are based on, for example, Buxton et al. [51].

### The NEO trial 2

The radial artery will be used as a mammario-radial graft (‘Y-graft’ where the radial artery is anastomosed end-to-side to the LIMA) or as an aorto-radial graft (free graft from the aorta) to the coronary artery of choice depending on which group the patient has been randomised into. The radial artery can be sequentially anastomosed onto multiple coronary arteries if so needed. If more grafts are needed than can be done using the radial artery, a piece of saphenous vein will be used to the additional grafts. The LIMA will almost always be used as the primary graft of choice.

### Description of graft choice

Patients with multi-vessel disease need more than the LIMA-LAD graft. The radial artery graft will be used to primarily left sided coronary arteries, i.e., the so-called diagonal and marginal branches. If a mammario-radial anastomosis is done it will also be possible to reach the end branch of the right coronary vessel named the posterior descending artery (PDA) with the radial artery graft. If an aorto-radial anastomosis is done and the patient has a significant stenosis on the right coronary artery it will not be possible to reach the PDA with the radial artery. Therefore, a piece of saphenous vein must be used as a graft to the PDA. In certain cases, where a significant left coronary main stem stenosis is present together with an occluded (i.e., >95% stenosis) right coronary artery, the patient will always receive a separate vein graft to the PDA. This is to secure the patient from only one inlet supplying the whole heart with blood and thereby endangering the patient should this trial show any problems with the mammario-radial anastomosis.

All patients will receive infusion with nitroglycerin intravenously for the first 24 postoperative hours and amlodipin (calcium antagonist) for 3 months according to department protocol.

### Assessment of outcomes

#### Overview of outcome measures

**Figure 2:**
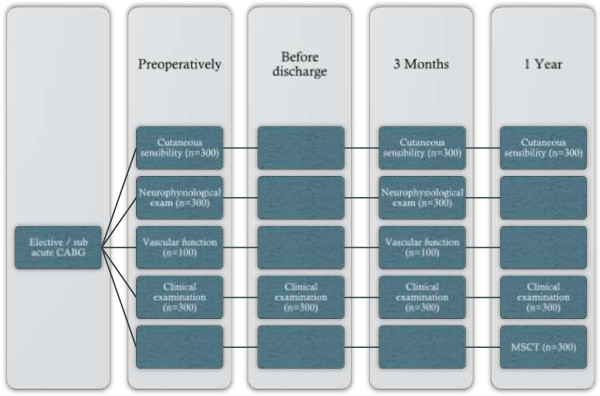
Examination flow chart.

As the trial is a 2 × 2 factorial designed trial, the outcomes are divided into two parts. All outcome measures are summarised in Table 2 and an overview of the time schedule for the individual participant can be seen in Figure 2.

**Table 2:** Measurements

	**Measurement time points**				**Data source**	**Blinding**
	**(x = measuring point, X = outcome endpoint)**					**(Y = yes, N = no)**
	**Preoperatively**	**Before discharge**	**3 months**	**1 year**		
Hand function questionnaire	x	x	X	x	Questionnaire	N
Neurophysiological examination	x		X		Datasheet	Y
Clinical neurological examination	x		X	x	Case report form (CRF)	N
Complication rate		x	X		Database	Y
Serious adverse events		x	x	X	Register	Y
Scar evaluation			x	X	CRF	N
Handgrip strength	x	x	x	X	CRF	Y
Muscle function	x	x	x	X	CRF	Y
Vascular function	x		X		Datasheet	Y
Graft patency ERAH vs. ORAH				X	Datasheet	Y
Pain scale (LANSS)	x	x	X	x	CRF	N
Cardiac or cerebrovascular events		x	x	X	Register	Y
Graft patency free radial artery vs. Y-graft				X	Datasheet	N
Cutaneous sensation for cold	x	x	X	x	CRF	N
Neuropathy screening (UENS)	x				CRF	N
Demographic baseline data	x				CRF	N

### Screening for polyneuropathy

All patients will undergo preoperative screening for polyneuropathy. This will be done by a clinical and neurophysiological examination using the Utah Early Neuropathy Scale (UENS) (Table 3) [52] including nerve conduction studies of the right peroneal and sural nerves. After statistical processing of the primary outcome in the part one objectives, the mentioned UENS will be used to control for a bias between the two groups (ERAH versus ORAH) as to predisposition to a peripheral nerve lesion from a pre-existing polyneuropathy after randomisation (confounder control).

**Table 3:** **Utah Early Neuropathy Scale (UENS)**[52]

**Motor examination:**		
0 = normal; 2 = weak		
Great toe extension	Left _____	Right_____
Total both sides (out of 4)		__________
**Pin sensation:**		
0 = normal		
1 for each segment with reduced sensation:	Left _____	Right_____
2 for each segment with absent sensation:	Left _____	Right_____
Total both sides (out of 24)		

**Allodynia or hyperesthesia:**		
0 = normal		
1 if present in toes or foot:	Left _____	Right_____
Total both sides (out of 2)		__________
**Large fibre sensation:**		
0 = normal; 1 = diminished; 2 = absent		
Great toe vibration:	Left _____	Right_____
Time:	Left _____ s	Right_____ s
Great toe joint position:	Left _____	Right_____
Total both sides (out of 8)		__________
**Deep tendon reflexes:**		
0 = normal; 1 = diminished; 2 = absent		
Ankle:	Left _____	Right_____
Total both sides (out of 4)		__________
**Total score (out of 42)**		__________

### The NEO trial 1: primary outcome

#### Hand function questionnaire (Table 4)

**Table 4:** **Hand function questionnaire**[53]

1. Right now, my hand and arm appear to be fine.	6. I am concerned about the appearance of my arm scar.
(1) Yes	(0) No scar at all
(2) No	(1) No concern
2. I feel pain in my arm or hand.	(2) Trivial concern
(1) No pain at all	(3) Mild
(2) Trivial	(4) Moderate
(3) Mild	(5) Quite concerned
(4) Moderate	(6) Very concerned
(5) Quite severe	(7) Extremely concerned
(6) Severe	7. My arm has a scar that causes discomfort.
(7) Severe, unbearable pain	(0) No scar at all
2. I feel numbness in my arm or hand.	(1) No discomfort
(1) No numbness at all	(2) Trivial discomfort
(2) Trivial	(3) Mild
(3) Mild	(4) Moderate
(4) Mode rate	(5) Quite uncomfortable
(5) Quite severe	(6) Very uncomfortable
(6) Severe	(7) Extremely uncomfortable
(7) Severe, unbearable numbness	8. I have difficulties with daily tasks because of the use of my hand and arm.
4. My arm or hand is swollen.	(1) No difficulties at all
(1) No swelling at all	(2) Trivial difficulties
(2) Trivial	(3) Mild
(3) Mild	(4) Moderate
(4) Moderate	(5) Quite marked
(5) Quite severe	(6) Very marked
(6) Severe	(7) Extremely marked
(7) Severe, unbearable swelling	Comments: ______________________
5. I have limited use of my hand.	9. Overall, my life is affected by the problems with my hand or arm.
(1) No limitations at all	(1) No worse at all
(2) Trivial	(2) Trivial life disruptions
(3) Mild	(3) Mild
(4) Moderate	(4) Moderate
(5) Quite severe	(5) Quite marked
(6) Severe	(6) Marked
(7) Extremely limited use	(7) Life radically worse
	Comments: ______________________

Sum score of hand function questionnaire items 2 through 8. The mean values in the ERAH group will be compared to the mean value in the ORAH group at 3 months after surgery.

The hand function questionnaire has been proposed by the RAPCO study as a tool to evaluate quality of life impact by radial artery harvest [53]. This study compared radial artery harvest to vein harvest. There has also been a quality of life study comparing ERAH versus ORAH which used a short questionnaire devised by Medalion as outcome [24]. The questionnaire used in the RAPCO study seems more precise than the four questions used by Medalion, which is why it has been chosen for this trial.

This outcome will be non-blinded. The trial nurse assigned to the NEO trial will record the responses of the participants. Source data will be the completed questionnaire.

### The NEO trial 1: secondary outcomes

#### Neurological deficits

The nerve function will be assessed in joint venture with the Department of Neurophysiology, Rigshospitalet, Copenhagen, Denmark, and is planned as follows:

1. Cutaneous sensibility on both forearms and hands by appraisal of dermatomes. All sensibility modalities are examined:

a. Cutaneous touch sensibility examined by von Frey filaments.

a. Deep pain sensibility examined by pressure algometry.

2. Sensory nerve conduction studies performed on both arms:

a. Median nerve (digit 2 – wrist); orthodromic technique.

a. Ulnar nerve (digit 5 – wrist); orthodromic technique.

a. Superficial sensory branch of the radial nerves (forearm – wrist); antidromic technique.

a. Lateral antebrachial cutaneous nerve (forearm – elbow); antidromic technique.

a. Medial antebrachial cutaneous nerve (forearm – elbow); antidromic technique.

3. Motor nerve conduction studies on both arms:

a. Median nerve (wrist – abductor policis brevis muscle (APB), elbow – APB).

a. Ulnar nerve (wrist – abductor digiti minimi muscle).

Neurophysiology technicians will perform the nerve conduction studies and the trial nurse will perform the quantitative sensory testing.

The outcome measure will be a composite of the 9 different tests performed. Even if one of these tests deteriorates significantly from preoperative values, the conclusion will be that neurological damage has taken place. The patients will undergo full examination preoperatively and at 3 months.

A significant clinical deterioration on the operated arm will be:

• von Frey filaments: measurement unit is milliNewton (mN). The filaments are individually calibrated to a tolerance range of ±10% of its nominal force. The normal set includes 12 filaments (corresponding to 12 successively increasing nominal values) in the range of 0.25 to 512 mN with the force increasing by a factor of two from filament to filament. A significant clinical deterioration is from one nominal value to ≥1 nominal value assessed as mean values at four sites on the hand and forearm.

• Algometry test: A pressure algometer (application surface 1 cm^2^) is placed over the palmar base of metacarpal II on the donor arm and pressure is applied until the pain threshold is reached. Corresponding control assessments are made contralaterally over the palmar base of metacarpal II. The test measuring unit is kiloPascal (kPa) and the test is validated for pain assessments over tender-points in normal muscles [54]. A significant deterioration is a change in pressure pain threshold (Δ[control arm – donor arm]) of ≥50 kPa (if the measurement is <150 kPa) or ≥100 kPa (if the measurement is ≥150 kPa).

• Sensory nerve conduction study: The peripheral nerves are activated using a surface stimulation electrode and the resulting sensory nerve action potential (SNAP) is recorded with surface electrodes over the nerve at a point more distal than or proximal to the stimulation point. The latency and amplitude of the SNAP is measured and the conduction velocity is calculated using the distance between stimulation and recording point. The results are compared to national multi-centre age-controlled normal values and reported as z-scores.

• Motor nerve conduction studies: The compound motor action potential (cMAP) is recorded from the muscle belly using surface electrodes in a pseudomonopolar configuration. The active electrode is positioned over the motor point of the muscle belly and the reference electrode is positioned at a nearby inactive site according to the laboratory procedures. The electrodes are readjusted to result in a cMAP with an initial negative deflection and a maximal amplitude. Responses from supramaximal stimulation at the wrist and in the median nerve also at the elbow are recorded. Distal motor latency and amplitude of the maximal cMAP is measured and motor nerve conduction velocity is calculated from the distance between stimulation sites. The results are compared to national multi-centre age-controlled normal values and reported as z-scores.

• In both sensory and motor nerve conduction studies, significant changes in amplitudes of the responses are defined as a reduction in z-score by a value of 2.

The outcomes will be non-blinded. The neurophysiology technician will not be blinded towards knowledge regarding the use of an endoscopic or open technique, since it will be evident by the scarring. Source data will be the standardised examination datasheet from the Department of Neurophysiology and the case report form (CRF) used by the trial nurse.

### Clinical neurological examination of both arms

• Change in cutaneous sensibility from baseline to the time point 3 months after surgery. Subjective changes in cutaneous sensibility will be registered using a map of the forearm and hand on which the participant can note changes coded by colour. The change values of the donor arm in the ERAH and ORAH group will be compared.

The trial nurse will, together with the participants, evaluate the change in cutaneous sensibility using a map of the forearm and hand to mark sensibility changes in the operated forearm and hand. Cutaneous sensibility will also be evaluated on non-donor arm to ensure test reliability and to test for mirror-defects post-surgery.

This outcome will be non-blinded. The trial nurse assigned to the NEO trial will be trained by the authors to perform the clinical neurological examinations. Source data will be the CRF used by the trial nurse.

### Complications in the donor arm

• Occurrence of complications at 3 months after surgery. Complications are defined as a composite of haematoma formation, wound dehiscence, or infection registered postoperatively (before discharge) and 3 months after the operation by the trial nurse.

This outcome will be non-blinded. The trial nurse assigned to the NEO trial will register the complications occurring in the operated sites of ERAH and ORAH participants. Source data will be database data from the surgical complication register.

### The NEO trial 1: exploratory outcomes

The following outcomes are ‘exploratory’, as we have not been able to perform power calculations due to none or very limited data from previous trials or studies.

### Serious adverse events

• Occurrence of the following serious adverse events at time point one year after surgery: reoperation for bleeding; revascularisation; myocardial infarction; stroke; or death (see section ‘Safety’).

This outcome will be blinded. The data will be collected directly from the Danish Patient Register using the participant’s civil registration number.

### Scar evaluation

• Stony Brooke Scar Evaluation Score [55] (Table 5) at one year after surgery. Comparison of mean scores in the ERAH and the ORAH group.

**Table 5:** **Stone Brook Scar Evaluation Scale**[55]

**Scar category**		**No. of point**
Width	>2 mm	0
	≤2 mm	1
Height	Elevated or depressed in relation to surrounding skin	0
	Flat	1
Colour	Darker than surrounding skin (red, purple, brown or black)	0
	Same colour or lighter than surrounding skin	1
Hatch marks or suture marks	Present	0
	Absent	1
Overall appearance	Poor	0
	Good	1

This outcome will be non-blinded. The trial nurse assigned to the NEO trial will be trained in and perform the scar evaluation. The data source will be the CRF.

### Handgrip strength

Maximal handgrip strength one year postoperatively (Table 6).

**Table 6:** Rating intervals of handgrip strength

**Rating**	**Males**		**Females**	
	**(lbs)**	**(kg)**	**(lbs)**	**(kg)**
Excellent	>141	>64	>84	>38
Very good	123–141	56–64	75–84	34–38
Above average	114–122	52–55	66–74	30–33
Average	105–113	48–51	57–65	26–29
Below average	96–104	44–47	49–56	23–25
Poor	88–95	40–43	44–48	20–22
Very poor	<88	<40	<44	<20

A hand dynamometer will be used to measure the maximum isometric hand and forearm muscle strength on both hands with the elbows flexed. Three consecutive tests will be done with 15 seconds of recovery time between tests; this test has been validated [56]. Measuring unit is kilograms and rating of the test is seen in Table 6.

This outcome will be semi-blinded. An independent health care professional who performs the handgrip strength measurements will be blinded by covering the skin of the donor arm to disguise the harvest technique used. The data source will be the CRF.

### Muscular function

• The following muscles will be rated according to the Oxford Scale for grading muscle strength (Table 7) at one year postoperatively: abductor pollicis brevis muscle; abductor digiti minimi muscle; 1st interosseus dorsalis muscle; flexor digitorum profundus muscle to finger 2 and 5; and extensor digitorum communis muscle.

**Table 7:** **Grading of muscle strength (Oxford Scale**)

Grade 0	No muscle movement
Grade 1	Muscle movement without joint motion
Grade 2	Moves with gravity eliminated
Grade 3	Moves against gravity but not resistance
Grade 4	Moves against gravity and light resistance
Grade 5	Normal strength

The rating scale is sometimes also referred to as ‘Medical Research Council Scale for Muscle Strength’. This outcome will be semi-blinded. An independent health care professional will perform the muscle strength evaluation blinded by covering the donor arm to disguise the harvest technique used. The data source will be the CRF.

### Hand function questionnaire single items

• Mean score of each of the hand function questionnaire items 2 through 8 (Table 4). The mean values in the ERAH group will be compared to the mean value in the ORAH group at 3 months after surgery.

### Neurological deficits single tests

• Occurrences of neurological deficits at 3 months after surgery. The deterioration of each of the clinical neurological tests is defined as a secondary outcome.

### MSCT evaluation of graft patency

• Patency of the graft at one year after the surgery. The patency will be divided into perfect patency, incomplete patency, string sign, and occluded according to assessment by MSCT, and the ERAH will be compared to the ORAH group.

The MSCT allows for assessment of cardiac structures and both 16-slice and 64-slice scanners have been validated for assessing the graft patency after CABG [57,58]. The patients included in the study are scanned using a 320-slice scanner (Toshiba Aquilion ONE, Japan). The scanning protocol is as follows: Gantry rotation time 350 ms, detector collimation 0.5 × 320. Tube voltage and current are chosen based on the patient’s body mass index ranging between 100 and 120 kV and between 280 and 500 mA. An intravenous contrast media (Visipaque 320 mg/mL, GE Healthcare, UK) is infused using a flow rate of 6 mL/s followed by a saline chaser. The contrast dye volume used is individually calculated according to patient body mass index (100–130 mL). Image interpretation is performed using commercially available software (Vitrea, version 3.0.1, Vital Images, USA). Grafts are evaluated by two experts in cardiac MSCT blinded to the patient’s randomisation status in the trial. MSCT is performed at the department of radiology one year after the operation.

This outcome will be blinded. Two employees from the Department of Cardiology and Radiology will do the interpretation of MSCT scans for patency, without knowledge of whether the radial artery has been harvested by endoscopic or open techniques.

### Neuropathic pain symptoms and signs

• The Leeds assessment of neuropathic symptoms and signs (LANNS) pain scale [59] at 3 months after surgery (Table 8). The Danish version of this assessment is tested to have a cut-off value at ≥12.

**Table 8:** **Leeds Assessment of Neuropathic Symptoms and Signs (LANSS)**[59]

**A. PAIN QUESTIONNAIRE**	
•	Think about how your pain has felt over the last week.
•	Please say whether any of the descriptions match your pain exactly.
1)	**Does your pain feel like strange, unpleasant sensations in your skin? Words like pricking, tingling, and pins and needles might describe these sensations**
	a. NO – My pain doesn’t really feel like this…………………………………………………………………..………………………………(0)
	b. YES – I get these sensations quite a lot……………………………………………………………….…….…………………..….………(5)
2)	**Does your pain make the skin in the painful area look different from normal? Words like mottled or looking more red or pink might describe the appearance.**
	a. NO – My pain doesn’t affect the colour of my skin……………………………………………………………………….….……………(0)
	b. YES – I’ve noticed that the pain does make my skin look different from normal………………….………………..…….…………….…(5)
3)	**Does your pain make the affected skin abnormally sensitive to touch? Getting unpleasant sensations when lightly stroking the skin, or getting pain when wearing tight clothes might describe the abnormal sensitivity.**
	a. NO – My pain doesn’t make my skin abnormally sensitive in that area….………………………………………………….……….……(0)
	b. YES – My skin seems abnormally sensitive to touch in that area………………………………………….…….……….……….….….…(3)
4)	**Does your pain come on suddenly and in bursts for no apparent reason when you’re still? Words like electric shocks, jumping and bursting describe these sensations.**
	a. NO – My pain doesn’t really feel like this…. ………….………………………………………………….….……………………………..(0)
	b. YES – I get these sensations quite a lot…………………………………………………………….…….…………………..…………….(2)
5)	**Does your pain feel as if the skin temperature in the painful area has changed abnormally? Words like hot and burning describe these sensations.**
	a. NO – I don’t really get these sensations………………………………………………….……….……………………..…………………(0)
	b. YES – I get these sensations quite a lot……………………………………………….……….………………………..………………….(1)
**B. SENSORY TESTING**	
Skin sensitivity can be examined by comparing the painful area with a contralateral or adjacent non-painful area for the presence of allodynia and an altered pin-prick threshold (PPT).	
1)	**ALLODYNIA**
	Examine the response to lightly stroking cotton wool across the non-painful area and then the painful area. If normal sensations are experienced in the non-painful site, but pain or unpleasant sensations (tingling, nausea) are experienced in the painful area when stroking, allodynia is present.
	a. NO, normal sensation in both areas………………………………………………….….………………………………......………………(0)
	b. YES, allodynia in painful area only………………………………………………………………….….………………….….….….………(5)
2)	**ALTERED PIN-PRICK THRESHOLD**
	Determine the pin-prick treshold by comparing the response to a 23 gauge (blue) needle mounted inside a 2 mL syringe barrel placed gently on to the skin in the non-painful and then in the painful areas.
	If a sharp pin-prick is felt in the non-painful area, but a different sensation is experienced in the painful area, e.g., none/blunt only (raised PPT) or a very painful sensation (lowered PPT), an altered PPT is present.
	a. NO, equal sensation in both areas…………………………………………………………………….….……………….….….….………(0)
	b. YES, altered PPT in painful area…………………….………………………………………….….…..….….……….….…..…………….….(3)
**SCORING:**	
Add values in parentheses for sensory description and examination findings to obtain overall score.	
**TOTAL SCORE** (maximum 24)…………….	
If score <12, neuropathic mechanisms are **unlikely** to be contributing to the patient’s pain.	
If score ≥12, neuropathic mechanisms are **likely** to be contributing to the patient’s pain.	

This outcome will be non-blinded. The trial nurse assigned to the NEO trial will record the responses of the participants. The data source will be the CRF.

### Vascular function

The vascular function will be assessed by single photon emission computed tomography (SPECT) of the hand. The technique used is ^99m^Technetium sestamibi imaging which is commonly known as a ‘MIBI scan’ and is most often used for myocardial perfusion [60]. ^99m^Technetium sestamibi is a lipophilic cation which when injected distributes accordingly to the blood perfusion. Using a gamma camera it is possible to register the gamma rays emitted by the decay of ^99m^Technetium sestamibi. When injected after stress exercise a possible insufficiently perfused area will be evident.

A total of 100 patients will be randomly included in the ^99m^Technetium sestamibi imaging examination of the hand; 50 patients will come from each of the ERAH and ORAH groups. There will be no consideration taken as to whether they are part of the aorto-radial or the mammario-radial groups. These patients will preoperatively and 3 months postoperatively be examined for vascular function in the donor hand comparing 3-month values to baseline values. Values will also be compared to the non-donor hand. The MIBI scan will show if there is any difference in perfusion after removal of the radial artery. There have been no previous studies examining the hand with MIBI scans, so the examinations aim to validate the technique in this setting. We propose that the ratio between measurements on thenar (base of the thumb) and on hypothenar (base of the little finger) can be a reliable indicator of hypoperfusion in the hand after removal of the radial artery.

Staff employed at The Department of Clinical Physiology, Nuclear Medicine, & PET, will perform the MIBI scans.

The outcome will be the quantitative difference in blood-flow to the hand, measured using the MIBI scan. Time point will be 3 months after surgery. The measuring unit is counts/cm^2^ that may be converted to estimate of Bq/cm^2^.

Since no previous similar test has been found in the literature search it is not possible to do pretrial power calculations.

This outcome will be blinded. The Department of Clinical Physiology, Nuclear Medicine, & PET will perform the exams and interpretations without knowledge of whether the radial artery has been harvested by endoscopic or open techniques. The data source will be a datasheet from the Department of Clinical Physiology.

### The NEO trial 2: primary outcome

#### Occurrence of cardiac and cerebrovascular events

• Occurrence of one of the following cardio- or cerebrovascular events: all-cause mortality, myocardial infarction, target vessel revascularisation, or stroke at one year postoperatively.

The outcome will be blinded. The data will be collected directly from the Danish Patient Register using the participant’s civil registration number. We are well aware of the exploratory nature of this outcome (please see below).

### The NEO trial 2: exploratory outcome

#### MSCT evaluation of graft patency

Same technique and procedure will be used as mentioned earlier in the text.

Outcome measure will be patency of the graft at one year after surgery. The patency will be divided into perfect patency, occluded, incomplete patency, and string sign according to assessment by MSCT.

The outcome will be non-blinded. Two employees from the Department of Cardiology and Radiology will do the interpretation of MSCT scans for patency, but it will be evident on the MSCT which proximal anastomosis site is used.

### Sample size

#### The NEO trial 1

We are planning a trial of a continuous response variable (hand function) from independent control and experimental participants with approximately one control per experimental participant. In a previous study, the response within each participant group was normally distributed with a standard deviation of 8 [53]. The targeted objective is, as mentioned, the hand function questionnaire (Table 4). If the true difference in the experimental and control means is 3, we will need to study 150 experimental participants and 150 control participants to be able to reject the null hypothesis that the population means of the experimental and control groups are equal with probability (power) 90%. The type I error probability associated with this test of this null hypothesis is 5%. In total, we thus need to include 300 participants.

#### The NEO trial 2

One case-control study found an event-free survival at 12 months of 97% using arterial revascularisation compared to 67% using venous revascularisation [47]. Another case-control study showed a survival free of cardiac event or death at 2.5 years of 11% for free grafts versus 17% for Y-grafts, but the risk of re-angina was 6.6% in the free graft group versus 4.6% in the Y-graft group [45]. No studies showed exactly what difference in occurrence of cerebrovascular composite outcomes could be expected between free grafts and Y-grafts, but a randomised clinical trial comparing arterial Y-graft with free saphenous vein grafts found significantly lower cardiac event-free survival at less than 2 years with a difference of about 20% events in free grafts versus 5% in Y-grafts [47]. Considering these studies, we expect maximally a difference in occurrence of cardio or cerebrovascular outcomes at one year postoperatively of 5% (15% in mammario-radial group vs. 10% in aorto-radial group).

We are planning a trial with approximately 150 experimental participants and 150 control participants. We will then only have a power of 25.7% to detect the difference of 15% composite outcomes in mammario-radial group versus 10% in aorto-radial group using a type I error probability of 5%. We will use an uncorrected *χ*^2^ statistic to evaluate this null hypothesis. Therefore, the NEO trial 2 will only be an exploratory trial to plan the size of a future randomised trial concerning the occurrence of cardiac and cerebrovascular events using mammario-radial versus aorto-radial anastomosis.

### Power estimations for secondary outcomes

#### Neurological deficits

Assuming a difference in occurrences of neurological deficits of 30% versus 15% in the two groups, using a type I error of 5% and by including 300 participants, we will have 88% power to detect a difference between the two groups.

### Clinical neurological examination of both arms

Assuming a difference in cutaneous sensibility of 30% versus 15% in the two groups, using a type I error of 5% and by including 300 participants, we will have 88% power to detect a difference between the two groups.

### Complications

Assuming a difference in complications of 7% versus 1% in the two groups, using a type I error of 5% and by including 300 participants, we will have 76% power to detect a difference between the two groups.

### Data collection

#### Method

Data will be collected in the CRF preoperatively and postoperatively at 3 months and 1 year after surgery. The CRF will be paper-based and will be entered into a digital database using OpenClinica software by two independent persons entering the same data independently. Data will be collected from interviews and examinations of the participants, from the surgical database (named ‘PATS’), from the electronic patient journal system (named ‘OPUS’), and from the Danish national patient database (named ‘Landspatientregistret’).

#### Timing

Before discharge, 3 months, and 1 year after the surgery, these patients will be clinically evaluated for a haematoma formation, infection, neurological deficits, and vascular dysfunction and their scar will be scored by a clinical examination. On the day before surgery and 3 months postoperatively, all patients will undergo a motor and sensory nerve conduction study. A subgroup of 100 patients will be selected randomly with 50% of patients in each of the ERAH and ORAH groups. This subgroup will undergo physiological examination of vascular function in the hand preoperatively and 3 months after surgery. Before including the first patients, a pilot-study of 5 patients undergoing physiological examination of vascular function will be done to evaluate examination technique implementation. In the vascular study group the opposite non-operated arm will act as control. MSCT will be conducted 1 year after surgery in all 324 patients with blinded evaluation of the secondary outcome (graft patency evaluation by 320 slice-MSCT). Figure 1 shows a flowchart over the randomisation procedure used in this trial.

See Table 2 for the planned collection of outcome data.

### Attrition

To avoid trial attrition we have chosen short-term outcome of one year. The close and personal contact with the trial nurse also lessens risk of loss to follow-up. Trial participants will find it beneficial to follow 3 month and 1 year postoperative visits for optimal treatment and controls not offered to non-NEO trial participants. The trial nurse will directly contact participants if they miss an outpatient visit. Likewise, the trial nurse will keep contact information of the participants up to date after every contact.

### Statistical methods

Statistical analyses will be conducted blinded by an independent statistician from the Copenhagen Trial Unit according to a detailed statistical analysis plan that will be developed before all the data are collected. The statistical analysis plan will thereby have been specified independently of the results. The statistical analysis will also take into account interventionist risk of bias.

The NEO trial 1 and the NEO trial 2 objectives do not overlap, so we do not need any adjustment for multiplicity and it will be possible to raise different conclusions for any one of the objectives.

The detailed statistical analyses will be described elsewhere. In brief, the analyses will be intention-to-treat. The primary analyses will, for all outcomes, be adjusted for the stratification variables (age and sex). Secondly, we will present unadjusted analyses.

### Analysis of continuous outcomes

Continuous outcomes will be described as mean, mean difference, median, standard deviation, and range. The general linear model (ANCOVA and ANOVA) will be used to compare the results between the intervention groups.

### Analysis of dichotomous outcomes

Dichotomous outcomes will be summarised as numbers, percentages, odds ratios, and 95% confidence intervals. Logistic regression will be used to compare the intervention groups.

### Non-parametric tests

The non-parametric van Elteren test will be used if the assumptions behind the parametric methods are not fulfilled.

### Threshold for significance

The thresholds for significance will be assessed according to the 5-point procedure suggested by Jakobsen et al. [61].

### Missing outcomes

If more than 5% of the primary and secondary outcomes are missing, multiple imputation will be used in the analysis of the primary and secondary outcomes (STATA 13). The imputation result will be considered the primary overall result.

### Screening for polyneuropathy

A statistical analysis comparing the UENS between ERAH and ORAH groups will be done. An analysis of the standardised amplitudes of motoric and sensory answers (z-scores) between the two groups will be diagnostic for polyneuropathy if two or more nerves (medianus, ulnaris, peroneus, suralis) have a z-score of less than 2.

### Ethics and trial registration

The trial protocol has been submitted to the Danish regional ethics committee for the Capital Region and has been approved under the case number: H-3-2012-116 in December of 2012. The planned data safety and registration procedures have been approved by the Danish Data Protection Agency under the case number: 2007-58-0015/30-0838. The trial is registered at ClinicalTrials.gov (Identifier: NCT01848886).

### Ethical justification

This trial will add to the evidence base regarding which ways to harvest radial artery grafts and which way to anastomose these grafts in the patients undergoing CABG. All patients will receive verbal and written information about the trial well before the operation is planned and they will all have signed an informed consent form before being included in the trial.

### Radiation exposure

• SPECT of the hands: Participants will be exposed to approximately 6 mSv corresponding to about 2 years of background radiation [62]. Two exams will be done resulting in 100 participants exposed to approximately 12 mSv of radiation or about 4 years of background radiation.

• MSCT: Approximately 7 mSv is the radiation exposure when doing the cardiac CT angiography. This corresponds to the same amount of radiation exposure when undergoing a classical angiography. All 300 participants will be exposed to this exam equivalent of less than 3 years of background radiation.

A participant can therefore at the most be exposed to 19 mSv of radiation corresponding to less than 7 years of background radiation; 100 participants will be exposed to the maximum radiation dose, since they are included in the SPECT group, but the remaining 200 participants will be exposed to 7 mSv, i.e., less than 3 years of background radiation.

The risk of cancer secondary to radiation exposure will, in the 100 participants (SPECT and MSCT), at the most rise from 30,000 deaths related to cancer to 30,095 deaths related to cancer per 100,000 persons exposed and for the remaining 300 participants (MSCT only) it will at the most rise from 30,000 deaths related to cancer to 30,035 deaths related to cancer per 100,000 persons exposed. These numbers are according to The Danish Board of Health [63].

### Pain and discomfort

• The neurophysiological examination can give rise to mild pain and some discomfort during the examination. The pain is expected to be visual analogue scale level 3 to 4.

Participants undergoing ERAH are expected to have less pain than ORAH [19]. Since ORAH is presently primary choice of radial artery harvest no participants in the trial will experience a higher degree of pain attributed to their inclusion in the trial.

## Discussion

The NEO trial will be able to compare the endoscopic with the open surgery radial artery harvesting techniques. Only three smaller randomised trials have been conducted trying to assess the patency and complications in ERAH versus ORAH. The NEO trial 1 will be able to assess if there is significantly less neurological complications when harvesting the radial artery with an endoscopic technique than by an open technique. We may find a tendency towards less haematoma formation and fewer infections in the ERAH than in the ORAH groups since it has been shown in other studies. No significant decrease in the vascular supply in the donor arms compared to non-donor arms has been indicated in previous studies, but the NEO trial 1 will try to assess if there could be a relative ischaemic state after radial artery harvest using a new diagnostic test. Particularly, any neurological deficits can limit the hand function of the patients postoperatively and it is important to try to get a clearer picture of this. The NEO trial 1 will also examine if there is any difference in patency in ERAH participants versus ORAH participants. This is an important point to be addressed since, if there is any difference, it can have severe consequences for the patients. All these points of investigation put together will determine the future of endoscopic harvest of the radial artery. We believe and hypothesise that the technique will be beneficial to the patients and will render the endoscopic procedure as the preferred technique when harvesting the radial artery for arterial revascularisation.

Another point of interest that the NEO trial 2 tries to enlighten is the optimal site of the proximal anastomosis when using the radial artery as a bypass graft. There are no randomised clinical trials telling us if aorto-radial or mammario-radial anastomosis is the best choice. Well aware that the NEO trial 2 does not have the power to show a significant difference when measuring major cerebrovascular events, we hope to get enough data to find a likely sample size for another randomised trial dedicated to this question. If this trial shows the mammario-radial technique being non-inferior to the aorto-radial technique, it will also be of great advantage. The mammario-radial technique has the great advantages of avoiding the side-clamp and the possibility to revascularise all three coronary vessels with a minimum of grafts required.

The NEO trial has the great advantage of using a 2 × 2 factorial design. This enables the trial to examine the benefits and harms of two different surgical strategies in the setting of one randomised clinical trial. The disadvantage of the trial is the exploratory nature when examining the two possible anastomotic sites of the radial artery. The complexity and elaborate neurological examinations including both objective tests and subjective answers on questionnaires is a major advantage of the NEO trial. This will make us able to both evaluate patient related parameters, such as quality of life, as well as factual complications comparing ORAH with ERAH. In an evolving field of modern cardiac surgery using arterial revascularisation and endoscopic technique, this trial will contribute with important facts necessary for optimising patient treatment.

### Limitations

There will be a partial lack of blinding since the surgeon and the patient cannot be blinded as to which operational technique has been used. All measurements available for blinding will be blinded. There will be an evaluator risk of bias in the part one objectives since the trial nurse cannot be blinded.

### Dissemination policy – trial results

All trial results and de-personalised individual participant data, whether positive, negative, or neutral, will end up in the public domain, preferably in peer-reviewed publications and in a public trial data repository. The trial is registered at clinicaltrials.gov, which ensures the results are public domain in accordance to the Consort Statement [64].

### Dissemination policy – authorship

Authorship will be determined according to the guidelines from the International Committee for Medical Journal Editors.

### Dissemination policy – reproducible results

The de-identified and de-personalised data will be uploaded to clinicaltrials.gov within 2 years after completion of follow-up of the last patient.

## Trial status

We started screening and randomising patients on the 16th of May 2013. By the 15th of March 2014 we have randomised and operated the first 50 patients and screened >1,000 patients.

## Abbreviations

CABG: Coronary artery bypass grafting surgery; cMAP: Compound motor action potential; CRF: Case report form; ECC: Extracorporeal circulation; ERAH: Endoscopic radial artery harvest; EVH: Endoscopic vein harvest; LAD: Left anterior descending artery; LIMA: Left internal mammary artery; MSCT: Multi-slice computed tomography; ORAH: Open radial artery harvest; PDA: Posterior descending artery; SNAP: Sensory nerve action potential; SPECT: Single photon emission computed tomography.

## Competing interests

The authors declare that they have no competing interest.

## Authors’ contributions

All authors made a substantial contribution to this manuscript and study design in regards to conception, design, and drafting. All authors have read and approved the final manuscript.
